# Feasibility of Imaging Esophageal Cancer with Labeled Somatostatin Analogue

**DOI:** 10.1155/2011/279345

**Published:** 2011-05-17

**Authors:** Gunnar Herlin, Lars Ideström, Lars Lundell, Peter Aspelin, Rimma Axelsson

**Affiliations:** ^1^Division of Medical Imaging and Technology, Department of Clinical Science, Intervention and Technology, Karolinska Institute, 141 86 Stockholm, Sweden; ^2^Department of Radiology, Karolinska University Hospital Huddinge, 141 86 Stockholm, Sweden; ^3^Department of Nuclear Medicine, Karolinska University Hospital, Huddinge, 141 86 Stockholm, Sweden; ^4^Division of Surgery, Karolinska Institute at Campus Huddinge, 141 86 Stockholm, Sweden

## Abstract

*Background*. While the surface of a cell normally has some amount of somatostatin receptors, these receptors are overexpressed to a very high degree in multiple neoplastic diseases. No data exist for esophageal carcinoma. *Purpose*. To find out whether esophageal carcinoma could be imaged using somatostatin receptor scintigraphy. *Material and Methods*. 34 patients with esophageal lesions were prospectively examined by ^99m^Tc-depreotide scintigraphy 2 and 4 hours after injection. Quantitative evaluation of ^99m^Tc-depreotide uptake was performed around the lesion (*T*) and in healthy lung parenchyma (*B*). The relative uptake was calculated as *T−B/B*. Scintigraphy results were compared with histopathology from surgery or biopsy specimens from endoscopic ultrasonography. *Results*. 21 patients had esophageal cancer, and 13 lesions were benign. Visual assessment revealed positive ^99m^Tc-depreotide uptake in 16 of the 21 cancers. The 13 patients without cancer had no depreotide uptake. The Mann-Whitney U test showed a statistically significant difference (*P* < .005) between ^99m^Tc-depreotide uptake in malignant and benign lesions, for both the 2-hour and the 4-hour measurements. *Conclusion*. Scintigraphic examination with ^99m^Tc-depreotide is feasible for imaging esophageal cancer, but the method is not suitable neither for screening or primary diagnosis, because of methods modest sensitivity. Our first results showed high specificity which should be used with caution, as the number of patients was relatively low. Further studies are needed to determine the role of the method.

## 1. Introduction

In Sweden, approximately 400 patients per year are newly diagnosed with cancer of the esophagus and an additional 200 with cancer of the cardia [1]. There are several methods to determine this diagnosis: computed tomography (CT), endoscopic examination of the esophagus (esophagoscopy) combined with endoscopic ultrasonography (EUS) and needle biopsy and positron emission tomography (PET) examination with ^18^F-Fluoro-deoxyglucose (^18^F-FDG).

CT has the disadvantage that it is less able to diagnose overgrowth of the tumor to adjacent organs and to detect small tumors <1 cm and growth to different layers in the esophageal wall. PET and the combination of ^18^F-FDG-PET/CT are both more accurate than CT alone with respect to diagnosing lymph node metastasis close to the tumor, and therefore staging [2]. EUS is superior to ^18^F-FDG-PET/CT with respect to diagnosing tumor growth through different layers in the esophageal wall, overgrowth to adjacent organs, and lymph node metastasis close to the tumor [2–5]. Examination with ^18^F-FDG-PET is based on the metabolic activity of the tumor, and reflects its pathophysiological characteristics. However, despite their high sensitivity in detecting esophageal cancer [2], these methods lack specificity; an elevated ^18^F-FDG uptake could be seen in different nonmalignant conditions, such as inflammation and Barrett's esophagus [6–12]. Another way to characterize tumors is evaluation of different receptors, such as the somatostatin receptors.

While the surface of a cell usually includes some amount of somatostatin receptors, these receptors are over-expressed to a very high degree in multiple neoplastic diseases such as neuroendocrine tumors [13] and in tumors of the central nervous system, breast, lung, and lymphoid tissue [12, 14].

When this study was initiated, there were several radiopharmaceuticals available for somatostatin receptor scintigraphy, including ^99m^Tc-depreotide. This tracer is ^99m^Tc-labeled, and demonstrates good imaging characteristics with a short investigation protocol. ^99m^Tc-depreotide has proven valuable in diagnosing pulmonary nodules [9–11]. We have previously used scintigraphy with ^99m^Tc-depreotide for the diagnosis of lung cancer, and showed an accurate discrimination between benign and malignant lesions with conventional gamma cameras [6, 9, 11]. There was a physiologically low ^99m^Tc-depreotide uptake in the thorax region. Therefore, visualization of overexpression of somatostatin receptors in cancer types other than lung cancer should, theoretically, be feasible. Moreover, our previous results showed that this tracer accumulates both in squamous cancers and in adenocarcinomas [15], which is of clinical relevance in view of the almost exponential increase in the incidence of adenocarcinoma in the distal esophagus. 

The aim of the present study was to find out whether esophageal cancer can be imaged scintigraphically with ^99m^Tc-depreotide and to determine the uptake characteristics of ^99m^Tc-depreotide in the two main cancer types of the esophagus and relate these to results in patients with benign lesions (Barrett's esophagus).

## 2. Material and Methods

The study was approved by the Regional Ethical Review Board in Stockholm, Sweden and the Radiation Safety Committee at Karolinska University Hospital, Huddinge.

### 2.1. Patients

34 patients with dysphagia were referred to the Surgery Department at Huddinge University Hospital and further examined with gastroscopy, EUS, and CT. Nine of these were female and 25 male, with a median age of 63-64 years (range: 33–85 years). Among the 34 patients, 21 had cancer of the esophagus and 13 had Barrett's esophagus. Five of the 21 patients with esophageal cancer had a Barrett's esophagus too. The cancer diagnosis was established by histopathological examination of biopsy specimens in 19 cases and with EUS and cytological confirmation of diagnosis in 2 cases. All patients with Barrett's esophagus were diagnosed via endoscope and subsequent multiple biopsies. 

Locoregional lymph nodes were evaluated with EUS and histological examination of surgical specimen.

### 2.2. Somatostatin Receptor Scintigraphy

^99m^Tc-depreotide (740 MBq) was administered via an antecubital vein. Single-photon emission computed tomography (SPECT) of the thorax was performed at 2 and 4 hours after injection, with the arms elevated, using three different gamma cameras. Most of the patients (25 of 34) were examined with a double-headed gamma camera (E-Cam, Siemens, Erlangen, Germany) and low-energy high-resolution parallel-hole collimators, using a 128 × 128 matrix, 64 projections through 360° rotation, and an acquisition time of 40 s per projection. An additional 5 patients were examined with a double-headed gamma camera (DST-XL; Sopha Medical Vision Scandinavia AB, Gif-sur-Yvette, France) and low-energy ultra-high-resolution parallel-hole collimators, using the same acquisition parameters as above. Finally, 4 patients were examined with a three-headed gamma camera (Picker IRIX, Cleveland, Ohio, USA) and low-energy high-resolution parallel-hole collimators, using a 128 × 128 matrix, 60 projections through 360° rotation, and an acquisition time of 64 s per projection. Transverse slices were reconstructed with an iterative algorithm (HOSEM v 3.5 iterative program; Hermes/NUD, Stockholm, Sweden) and formatted as a 128 × 128 matrix without attenuation correction. Images were postfiltered with a three-dimensional Fourier filter (Butterworth filter) with a cut-off frequency of 1.1 cycles/cm (order 5.00).

**Figure 1:**
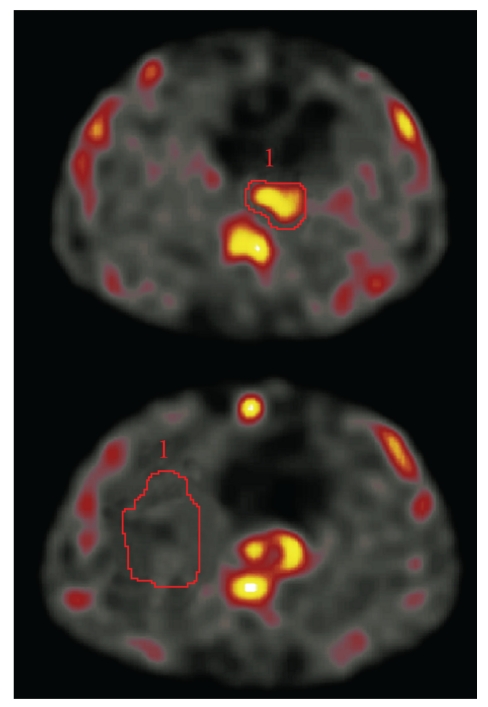
Evaluation of scintigraphic images with ^99m^Tc-depreotide. Region of interest (ROI) was drawn manually around the esophageal tumor on each slice, using small margins, and a background ROI was drawn in healthy lung parenchyma.

The results were evaluated both through visual assessment and through quantitative calculations in the 2-hour and 4-hour images. CT scans were used for an accurate localization of the ^99m^Tc-depreotide uptake and for placement of the region of interest (ROI). On visual assessment, any focal ^99m^Tc-depreotide uptake in the region of the known esophageal lesion was considered pathological. The quantitative evaluation of ^99m^Tc-depreotide uptake was performed retrospectively on SPECT images in all 34 patients. First, an ROI was drawn manually around the esophageal tumor on each slice, using small margins. Next, a background ROI was drawn in healthy lung parenchyma (Figure 1). A volume of interest (VOI) was obtained by adding all ROIs. In-house software, originally developed for volumetric measurements in magnetic resonance images and implemented on a Hermes workstation (Hermes Medical Solution AB, Stockholm, Sweden), was used to calculate the total counts and volume of the tumor and background VOIs, thus giving a count density (counts/cm^3^). To produce a normalized tumor uptake, each patient was normalized to his or her own normal lung parenchyma using the formula *U* = (*T* − *B*)/*B*, where *U* is the normalized uptake, *T* is the count density in the tumor, and *B* is the count density in the lung parenchyma. To increase accuracy and to investigate the intraobserver variability, evaluations were performed twice, 6 months apart, by the same radiologist and the mean value of the two uptake values was used in further analysis. In addition, a second radiologist made individual evaluations in order to investigate the interobserver variability of the uptake values at 2-hour images.

### 2.3. Statistics

For patients with negative uptake (i.e., tumor count density lower than the lung background count density), the uptake was scored as zero. Due to the small number of patients in each group, a nonparametric test was chosen. A two-sided Mann-Whitney *U* test was used to investigate the difference in uptake between malign and benign tumors. To assess intraobserver and interobserver variability, intraclass correlation coefficients (ICC) were determined [16]. All statistical analysis was performed in Statistica 9.0 (StatSoft. Inc, Tulsa, OK, USA). Data were analyzed based on both the 2-hour and the 4-hour postinjection recordings except for the ICC which was only determined for the 2-hour recordings. A difference in uptake was considered significant if the *P* value was less than  .05.

## 3. Results

Among the 21 patients with cancer of the esophagus, 8 had squamous cell carcinoma, 11 had adenocarcinoma, 1 had an undifferentiated cancer, and 1 had an intramucosal cancer. Tumor size varied from 5 mm to 11 cm. The position of the tumor was in the proximal esophagus in 2 cases, in the middle part in 5 cases, and in the distal part in the remaining 14 cases.

 Visual assessment revealed a pathological ^99m^Tc-depreotide uptake in 16 of the 21 cancer patients (true-positive 76%) and an absence of pathological uptake in the remaining 5 (false-negative 24%). Six of the eight patients with squamous cell carcinoma and nine of the eleven patients with adenocarcinoma showed a pathological ^99m^Tc-depreotide uptake. The remaining patient with pathological uptake had an 11 cm undifferentiated cancer in the mid-esophagus with a very high ^99m^Tc-depreotide uptake. Among the false-negative cases, one had a small (5 mm) squamous cell cancer located in the middle part of the esophagus. The remaining four undetectable cancers were above 1 cm in size (varying from 12 × 9 mm to 12 × 38 mm) and were located in the distal part of the esophagus. The details of all recordings are given in Table 1. The sensitivity of ^99m^Tc-depreotide scintigraphy in the detection of esophageal cancer was thus 0.76 95% confidence interval 0.55 to 0.89. 

**Figure 2:**
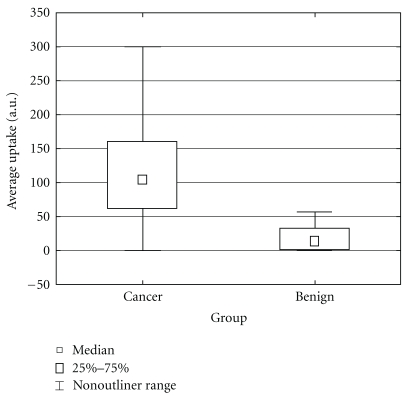
^99m^Tc-depreotide uptake measured 2 hours after injection in patients with esophageal cancer and Barrett's esophagus.

There was no ^99m^Tc-depreotide uptake in the columnar metaplastic mucosa in any of the 13 Barrett's patients, irrespective of the presence of low and high-grade dysplasia in the metaplastic epithelium (Figure 2). The specificity of ^99m^Tc-depreotide scintigraphy in this cohort of patients was thus 1.00, 95% confidence interval 0.77 to 1.00. 

There were no significant differences between the ROI delineation and quantitative measurement of ^99m^Tc-depreotide performed on the 2-hour acquisitions and those performed on the 4-hour acquisitions. A corresponding second ROI delineation and quantification, performed 6 months later, gave consistent results. Both intraobserver and interobserver variability was low with ICC = 0.97 when comparing the evalutions by the same radiologist (intraobserver) and ICC = 0.96 when comparing the evaluations made by the two radiologists (interobserver).

A statistically significant difference (*P* < .005) was found between ^99m^Tc-depreotide uptake in malignant lesions compared to that in benign or premalignant lesions (Figure 2), both 2 and 4 hours after injection. The absolute ^99m^Tc-depreotide uptake value was also higher in all malignant lesions after 2 compared to 4 hours. There was no difference in uptake between adenocarcinoma and squamous cell carcinoma. 

In the 13 patients who had lymph node metastases at the final examination of the surgical specimen and with EUS only 5 showed ^99m^Tc-depreotide uptake in the area of the lymph nodes.

## 4. Discussion

In this study, we have shown that the imaging of esophageal cancer by means of somatostatin receptor scintigraphy with ^99m^Tc-depreotide is feasible. Our hypothesis was based on two facts: first, that the physiological ^99m^Tc-depreotide uptake in the thorax is low. Therefore, this could be a suitable area for tumor detection in most cases, and, second, that esophageal cancer has the same main histopathological types as lung cancer such as adenocarcinoma and squamous cell carcinoma. As scintigraphy with ^99m^Tc-depreotide is useful for lung cancer detection, this second fact suggested that it could also be applied in esophageal cancer. 

The majority of tumors (16/21) displayed a significant uptake of the tracer which could be clearly distinguished from that in the surrounding tissue. It was not unexpected that tumors under or near 10 mm in size were missed on the scintigraphic images. The detection limit of conventional gamma camera due to poor spatial resolution is well known, and according to widespread consensus scintigraphic methods are not suitable for screening purposes for any cancer types. Another observation is that even larger tumors in the distal part of the esophagus, 4 of 13 in the present study, could be missed with this method. Uptake of ^99m^Tc-depreotide in lung cancers located in the lowest part of the right low lobe [6] and even in esophageal cancers located at the level of the diaphragm and lower in the abdomen could be obscured because of the high physiological tracer uptake in the liver.

Our sensitivity figure of 76% is only an approximate value, due to the small number of patients in this study. Still, this is somewhat lower than both the sensitivity for detecting lung cancers [2, 6–11] with the same tracer and that for detecting lung cancer with ^18^F-FDG-PET [6–8]. While ^18^F-FDG uptake reflects a metabolic activity of the lesion and is not specific to tumors [6–8], overexpression of somatostatin receptors on tumor cells could give another valuable piece of information regarding tumor properties.

As a control group, we used patients with Barrett's esophagus. Barrett's esophagus refers to an abnormal change (metaplasia) in the cells in the lower end of the esophagus. It is thought to be caused by damage from chronic acid exposure or reflux oesophagitis. This metaplasia confers an increased risk of adenocarcinoma. None of the 13 cancer-free Barrett's esophagus patients in this study showed an increased ^99m^Tc-depreotide uptake. Meanwhile, only 3 of 5 patients with both cancer and Barrett's esophagus showed an increased ^99m^Tc-depreotide uptake, leaving 2 false-negative results. The specificity of 100% for the applied method is high but should be used with caution, as the number of patients was relatively low and the spectra of different benign conditions in the esophagus was not fully represented in this pilot study.

Our results in the detection of loco-regional lymph node metastases were unsatisfactory. Only 5 of 13 patients with metastases seen with EUS and confirmed by histological examination were clearly detected by ^99m^Tc-depreotide scintigraphy. It is probably caused by the close location to the primary tumor, where a high depreotide uptake cannot be separated from the uptake in the metastatic lymph nodes. It was disappointing to note that very few of the local, metastatic lymph nodes could be detected by this method. Through this pilot observation it can be envisioned that this technology cannot add to the available methods, such as EUS, in determining the node status in oesophageal cancer during the diagnostic and therapeutic workup. 

 As this study is the first of its kind, we considered it important to explore whether the quantitative assessment was reliable between different investigators and over time. Both intraobserver and interobserver variability was very low meaning that the applied calculations have good reliability. 

 We applied a somatostatin receptor scintigraphy with ^99m^Tc-depreotide in a previously nonexplored cancer type where the optimal acquisition time was unknown. We used the same starting point for the imaging session as for the standard procedure in the detection of lung cancer, that is, 2 hours after injection [8, 10, 11]. During the last decade there has been a trend of performing a double-phase registration in order to increase specificity. The double-phase registration is based on assumptions that the relative tracer uptake in benign lesions decreases with time, while uptake in malignant lesions remains high or even increases with the time [10, 17–20]. This approach is now in routine for parathyroid scintigraphy, and its use has also been suggested for scintimammography, tumor imaging with ^18^F-FDG-PET, and somatostatin receptor scintigraphy with ^99m^Tc-depreotide in lung cancer patients [17–20]. In order to optimize the imaging procedure from the very beginning, we performed double-phase registrations with imaging 2 and 4 hours after injection. Our results showed that in the majority of patients the absolute uptake decreased more rapidly in the background (i.e., the normal lung parenchyma compared to the malignant lesions in the esophagus), resulting in a higher relative uptake in cancer over time. As both the 2-hour, and the 4-hour quantitative evaluations showed a statistically significant difference (*P* < .005) between ^99m^Tc-depreotide uptake in malignant lesions and in lesions without cancer, the 4-hour imaging seems unnecessary, and thus could be omitted for practical reasons. 

 Although future immunohistochemical studies will be needed to carefully map the density, detailed distribution, and localization of somatostatin receptors in the squamous cell esophageal carcinoma and adenocarcinomas, our data indicate that there is no major difference as reflected by the similarity in tracer accumulation between these two major tumor types. It is, however, of particular interest that in patients with Barrett's esophagus, no accumulation of tracer was observed either in those with or in those without dysplastic histomorphologic changes in the columnar epithelium. Since there is no corresponding preneoplastic condition, concerning the squamous cell carcinoma development, it can be hypothesized that the somatostatin receptor expression reaches far higher levels in infiltrative neoplastic growth than in the intraepithelial neoplastic disease states. If so, this observation may be potentially very important and offer unique clinical opportunities, for example, when PET/CT or somatostatin receptor scintigraphy with ^99m^Tc-depreotide technologies are applied. 

Results of this pilot study showed feasibility of imaging oesophageal carcinoma with labeled somatostatin receptor analogue. We used a single-photon emitting tracer, but the results should be applicable or even better with positron emitting tracers. Use of PET/CT cameras combines a better spatial resolution of functional PET imaging with detailed anatomical information leading to a higher sensitivity. A multitude of new PET analogs are applied, whereas [^68^Ga-DOTA^0^, Tyr^3^] octreotide [21] or [^68^Ga-DOTA^0^, Tyr^3^] octreotate [22] is likely to become the new standard for somatostatin receptor imaging with PET. This is because these analogs have a high affinity for the somatostatin receptor subtype-2 and because ^68^Ga is a generator product with a relatively simple labeling [23]. The additional reason is that their ^90^Y- or ^177^Lu-labeled counterparts are used for Peptide Receptor Radionuclide Therapy (PRRT) and it seems desirable that peptide used in diagnostic imaging mimics the peptide used later for therapy.

After the successful visualization of somatostatin receptor positive tumors, a logical next step would be to use radiolabelled somatostatin analogues as a treatment of these patients. Such attempts were undertaken in patients with inoperable and/ormetastatic neuroendocrine tumors. While the objective responses for chemotherapy with the median time to progression is reported to be less than 18 months, PRRT with ^90^Y-octreotide or ^177^Lu-octreotate performs considerably better with a median time to progression of 30 and 40 months, respectively [23], and significantly improved quality of life [24].

What could the future clinical application of our results be? Obviously, the method is not suitable neither for screening or primary diagnosis, because of methods modest sensitivity. Could this method be used for the detection of distant metastases expressing somatostatin receptors with somatostatin receptor-mediated radionuclide therapy as a consequent result? Does the uptake of the ^99m^Tc-depreotide could be related to the prognosis of the oesophageal tumor? May this method be used in evaluation of treatment response in patients with tracer uptake? Does the natural history of Barrett's esophagus and its malignisation could be predicted by tracer uptake? These issues are still to be answered.

### 4.1. In Conclusion

Scintigraphic examination with ^99m^Tc-depreotide is feasible for imaging esophageal cancer, but the method is not suitable neither for screening or primary diagnosis, because of methods modest sensitivity. Our first results showed high specificity which should be used with caution, as the number of patients was relatively low. Acquisitions starting 2 hours after injection are optimal and suffice for imaging. Further studies are needed to explore and determine the role of somatostatin receptor scintigraphy in clinical practice.

## Figures and Tables

**Table 1:** Tumor type, size, and location, CT result, and ^99m^Tc-depreotide uptake in 21 esophageal cancer patients.

*N*	Diagnosis	Tumor size in mm	Location	CT	^99m^Tc-depreotide
1	Sqcc	5	Middle	Neg.	Neg.
2	Ac	17 × 45	Distal	Pos.	Pos.
3	Sqcc	55 × 40	proximal	Pos.	Pos.
4	Ac in B.	65 × 55 × 13	Distal	Pos.	Pos.
5	Ac	90 × 75 × 25	Distal	Pos.	Pos.
6	Imc and B.	12 × 9	Distal	Neg.	Neg.
7	Sqcc	30 × 10	Middle	Pos	Pos
8	Ac	20 × 90	Middle	Pos.	Pos.
9	Ac	60 × 25 × 9	Distal	Pos.	Pos.
10	Sqcc	50 × 45	Distal	Pos.	Pos.
11	Small cell cancer	110 × 24	Middle	Pos.	Pos.
12	Ac in B	60 × 65	Distal	Pos.	Pos.
13	Ac	25 × 15	Distal	Neg.	Pos.
14	Ac in B	20 × 25	Distal	Pos.	Pos.
15	Sqcc	60 × 10	proximal	Pos.	Pos.
16	Sqcc	15 × 55	Distal	Pos.	Pos.
17	Ac	15 × 50	Distal	Pos.	Pos.
18	Ac	15 × 15	Distal	Neg.	Neg.
19	Ac in B	20 × 25	Distal	Neg.	Neg.
20	Sqcc	14 × 5	Middle	Neg.	Pos.
21	Sqcc	23 × 38 × 12	Distal	Pos.	Neg.

Ac: adenocarcinoma; Sqcc: squamous cell carcinoma; B: Barrett's esophagus; Imc: intramucosal cancer. ^99m^Tc-depreotide uptake classified as negative or positive based on visual assessment. CT pos.: tumor is visible on the CT images; CT neg.: tumor is not visible on the CT images.
